# Cutaneous signs of insulin resistance with central obesity: insights into adipocentric metabolic dysfunction in South Asians

**DOI:** 10.3389/fcdhc.2025.1691675

**Published:** 2025-12-03

**Authors:** Aditya Saxena, Pradeep Tiwari, Anamika Gora, Balram Sharma, Rajendra Mandia, Shalu Gupta, Anurag Dhakar, Ravinder Kumar Lamoria, Praveen Choudhary, Sandeep Kumar Mathur

**Affiliations:** 1 Department of Bioinformatics, Faculty of Engineering and Technology, Marwadi University, Rajkot, India; 2 Department of Biotechnology and Bioinformatics, Birla Institute of Scientific Research, Jaipur, India; 3 Department of Endocrinology, Sawai Man Singh Medical College and Attached Hospital, Jaipur, India; 4 Department of General Surgery, Sawai Man Singh Medical College, and Attached Hospital, Jaipur, India; 5 Department of Orthopedics, Sawai Man Singh Medical College, and Attached Hospital, Jaipur, India

**Keywords:** Acanthosis Nigricans, acrochordon, Asian Indians, central obesity, insulin resistance & clustering diseases (metabolic syndrome), adipose tissue molecular pathology & mechanisms, insulin resistance, metabolic syndrome

## Abstract

**Aim and objectives:**

To investigate the *C*utaneous *S*igns of *I*nsulin *R*esistance, namely acanthosis nigricans (AN) and acrochordon (AC), in individuals with *C*entral *O*besity (CO-CSIR) as physical predictors of metabolic syndrome (MetS), underlying adipose tissue pathology, and consequent pathophysiological traits in South Asians.

**Methods:**

In this study, 371 participants were recruited in a tertiary care facility and grouped based on the presence of cutaneous signs (AC and/or AN) and central obesity. Each participant was assessed for MetS, T2D, as well as other demographic, biochemical, and radiological parameters. Additionally, we conducted transcriptome profiling in adipose depots for selected individuals to investigate whether there are modules of co-expressed genes that show a correlation with cutaneous sign(s) and MetS/T2D, in order to decipher the link between these signs and metabolic derangement.

**Results:**

ANOVA analyses revealed significant differences among groups with varying cutaneous signs and W:H ratios, particularly highlighting the combined predictive capability of these markers. *Post hoc* tests further confirmed these findings, showing substantial differences in MetS, T2D, and HOMA-IR between these groups. Sensitivity-specificity analyses demonstrated that CO-CSIR provides a more balanced and accurate prediction of MetS status compared to either CO or CSIR alone. Furthermore, in predicting MetS status based on the number of MetS components (from 5 to ≥1), it also performed well. WGCNA analysis in visceral fat revealed modules of co-expressed genes significantly correlated with AC and MetS, indicating a link between the adipose tissue molecular pathology and the cutaneous signs.

**Conclusion:**

CO-CSIR is a promising physical sign for predicting MetS and the underlying adipose tissue-driven dysmetabolism in South Asians.

## Introduction

The traditional concept of metabolic syndrome (MetS) as an atherosclerotic cardiovascular risk prediction (CVD) tool has long been debated, and till now it remains not universally accepted as a clinical entity (1–3). Moreover, the consensus terminology, which could precisely define this adipocentric disease cluster also remains uncertain. Several names have been suggested including insulin resistance syndrome (4), syndrome X (4), Reaven syndrome (5), cardiometabolic syndrome (6), multimorbidity disease (7), and the latest term, multiple long-term conditions (MLTC) (7).

Several recent investigations have shed light on its underlying pathophysiological mechanisms and based on their findings, MetS can be redefined as “a state of metabolic dysregulation characterized by insulin resistance (IR), hyperinsulinemia, and a predisposition to ASCVD, hypertension, Alzheimer’s disease, certain cancers, and many other disorders” (8–10). Additionally, individuals with MetS may present with low-grade inflammation, oxidative and endoplasmic reticular (ER) stress, mitochondrial dysfunction, and impaired exercise capacity (11–13). This pathophysiology is usually associated with obesity or overweight, but may also involve subtle excess adiposity such as ectopic fat deposits in the liver, muscles, and pancreas (14–16). In other words, MetS cannot be equivaled with simple obesity measured as body mass index (BMI). Obesity may not always cluster with IR and the clustering diseases, particularly in metabolically health obese persons (MHO). The other facet of the coin is that the current insight into its pathophysiology, the classical concept of MetS (so called “MetS clinical” for the purpose of this article) can be considered clinical manifestation of a primarily an adipocentric metabolic disorder, which indirectly affects several metabolically active organs and vascular system (so called “MetS disease or adiposopathy” for the purpose of this article) (8). This disease concept for MetS is particularly relevant for South Asians, who for a given BMI show relatively high IR and higher prevalence of the clustering diseases. Moreover, they cluster with central rather than generalized obesity (17–19). This adipocentric disease concept of “MetS disease or adiposopathy” in South Asians has recently been investigated by us. We find that it is ectopic liver fat rather than the intraperitoneal fat surrounding the visceral organs that show a stronger association with IR. Moreover, in the higher quartile of IR, this correlation becomes stronger (20). In diabetics, both abdominal and peripheral adipose depots exhibit molecular features of pathological adipose tissue (i.e. adiposopathy). Its molecular pathways also converge on the processes of adipogenesis and inflammation (i.e., the cardinal features of pathological adipose tissue). Additionally, several modules of co-expressed genes in adipose tissue show association with various intermediate phenotypic traits of T2D and MetS, for example adipocyte size, insulin resistance, β-cell function, and circulatory adipocytokine levels etc. (21–23). Several regulatory non-coding RNAs, transcription factors, and kinases that are imputed to regulate pathological transcriptomes in the IR state are mapped to several genomic loci showing association with T2D in genome-wide association studies (GWAS) (24). The metagenome of the transcriptome of various tissues including, adipose tissue of IR individuals, deduced by a machine learning model, predicted the T2D with approximately 73% accuracy (25). In summary, all these observations suggest that IR, T2D and other clustering diseases are associated with pathological changes in adipose tissue characterized by a distinct molecular signature at transcriptomic level. In other words, these findings support the concept of an adipocentric complex genetic disease (the so called “MetS disease or adiposopathy”) for IR, T2D and other clustering diseases. Considering the magnitude of the problem and the increased risk of vascular-metabolic morbidity and mortality, diagnosing “MetS disease or adipsopathy” and its underlying apocentric metabolic dysfunction in clinical practice as well as at the community level is crucial. As mentioned previously, several clinical definitions and diagnostic criteria of “MetS clinical” have been recommended by different professional organizations, but none is universally accepted (26–32). An alternative strategy could be to predict MetS (“MetS clinical”) as well as and the underlying adipocentric metabolic dysfunction (“MetS disease or adipsopathy”) using physical signs. Several physical signs are associated with IR, T2D and other clustering diseases. Anthropometric parameters like BMI, waist circumference (WC), and the Waist-to-Hip (W:H) ratio estimate body fat content and its distribution in central versus peripheral compartments.

In addition to anthropometric measures like BMI, waist circumference, and W:H ratio, two cutaneous signs of IR—acanthosis nigricans (AN) and acrochordon (AC)—may serve as bedside predictors (33, 34). AN is characterized by hyperpigmented, velvety plaques that typically occur in the intertriginous areas such as the neck, axillae, groin, and other body folds. AC, commonly known as a skin tag, is a benign, pedunculated (having a stalk) lesion that commonly occurs in areas where the skin folds or creases, such as the neck, armpits, groin, and eyelids.

Several previous studies have reported these cutaneous signs show an association with IR and clustering diseases (i.e., MetS clinical) in South Asians (35–43). However, few studies have demonstrated the clinical importance of these cutaneous signs as bedside predictors of the IR and clustering diseases, particularly in context to the anthropometric physical signs of central obesity (44). Our hypothesis is that the presence of these cutaneous signs, particularly in individuals having central adipose tissue deposition, can serve as a simple clinical tool to predict IR, clustering diseases and its underlying adipocentric metabolic dysfunction (“MetS clinical” as well as “MetS disease or adiposopathy”). Therefore, in the present study, we explored the utility of these cutaneous signs of IR in predicting “MetS clinical” and the underlying adipocentric metabolic dysfunction (i.e., “MetS disease or adiposopathy”) in South Asian, particularly in context of clinical measures of central obesity (CO) assessed by the W:H ratio (i.e. CO-CSIR).

Additionally, we conducted a gene expression microarray analysis and profiled gene expression in three adipose tissue depots: abdominal subcutaneous, visceral, and peripheral subcutaneous. We used a systems biology method, WGCNA, to determine if signs of CO-CSIR could show an association with any module of co-expressed genes, as they might point toward some underlying molecular pathology of adipose tissue.

## Material and methods

### Study overview

This cross-sectional analysis was conducted on the data of 371 individuals (aged > 25 years; 210 M: 161F) attending Sawai Man Singh (S.M.S.) Medical College Hospital and participated in a series of research projects assessing adipose tissue dysfunction underlying T2D and shared a common clinical, biochemical, and radiological evaluation protocol. However, adipose tissue biopsies of molecular investigations were taken from the individuals undergoing either abdominal or femur bone surgery for non-infectious or malignant conditions like hernia, cholecystectomy, or trauma in case of femur bone surgery. These studies were approved by the institutional ethics committee of S.M.S. Medical College, and all participants provided informed written consent. The exclusion criteria were the presence of infection, malignancy, or the use of drugs affecting body fat, such as thiazolidinedione and glucocorticoids.

For the present study, participants were assigned into groups based on the number of criteria they met out of the five recommended by the National Cholesterol Education Program Adult Treatment Panel III (NCEP ATP III) for clinical diagnosis of Metabolic Syndrome (MetS): increased waist circumference (≥90 cm for men and ≥80 cm for women), elevated triglycerides (≥150 mg/dl or current drug treatment for elevated triglycerides), low HDL cholesterol (<40 mg/dl for men, <50 mg/dl for women, or current drug treatment for low HDL cholesterol), hypertension (blood pressure ≥130/≥85 mmHg or current drug treatment for hypertension), and elevated fasting glucose (≥100 mg/dl or current drug treatment for elevated fasting glucose). Participants were classified as meeting each criterion either through direct clinical measurement or current pharmacological treatment, in accordance with NCEP ATP III guidelines. We acknowledge that medication use may influence biomarker levels and have noted this as a limitation of the study.

Would you like help drafting the exact revision text for your manuscript or integrating this into your point-wise response table?

All the subjects underwent comprehensive clinical evaluation, including detailed clinical history and physical examination including cutaneous signs of insulin resistance and anthropometric measurements (height, weight, BMI, waist circumference (WC), hip circumference (HC), and W:H ratio. Supine blood pressure was measured using a mercury sphygmomanometer (BPMR-120 Diamond deluxe, Industrial Electronics and Allied Products, Maharashtra, India) after 10 min of rest. During physical examination, the presence of AN and AC was diagnosed based on specific clinical criteria. The diagnostic criteria for AN included the presence of thick, rough, irregular wrinkles and brown pigmentation of the skin, which commonly affect the neck and armpits. The diagnostic criteria for AC included the presence of small, soft, and pedunculated protrusions commonly affecting the neck and armpits.

### Anthropometric measurements

Weight and height were measured using a standard balance beam scale and stadiometer, respectively. Body mass index was calculated from the ratio of body weight in kg to height in meters squared and expressed as kg/m^2^. Waist circumference was measured using a non-stretchable flexible tape at the site of maximum circumference midway between the lower ribs and anterior–superior iliac spine. Hip circumference was measured over the greatest protrusion of the gluteal muscles.

### Biochemical investigations

A venous blood sample was obtained after an overnight fast of at least 8 h. Biochemical measurements including fasting blood glucose (FBG) and lipid profiles including total cholesterol (TC), triglycerides (TG), high-density lipoprotein cholesterol (HDL-C), and very low-density lipoprotein (VLDL) were performed using a Kopran AU/400 (Olympus corporation, Shinjuku, Tokyo, Japan) fully automated analyzer. Serum insulin was measured using a chemiluminescent immunometric assay (Immulite 2000 machine, Siemens Healthineers AG, Erlangen, Germany). HbA1c was measured by turbidimetry method using BioSystems (Biosystems, S.A. Barcelona, Spain) kits. HOMA-β was calculated using the following formula (28).


$$\frac{360\;x\;[Insulin\;in\;µU/ml]}{[Glocose\;in\;mg/dL]-63}$$

HOMA-IR is a measure of insulin resistance and is calculated using the following formula (28):


$$\frac{\left[Glucose\;in\;mg/dL]\;x\;[Insulin\;in\;µU/ml\right]}{405}$$

### Definition and diagnostic criteria for metabolic syndrome

#### Radiological investigations

Abdominal fat content and distribution among the visceral adipose tissue (VAT), subcutaneous adipose tissue (SAT), and ectopic hepatic compartments were estimated by magnetic resonance imaging. The MRI scans were conducted at S.M.S. Hospital in the Department of Radiology, using a 3-T Philips Ingenia Machine. A single investigator interpreted the scans obtained using Osirix, remaining unaware of the clinical status of the study subjects. A single scan (3 mm) of the abdomen was performed at the level of the L4-L5 vertebrae and analyzed for the cross-sectional area of adipose tissue, expressed in square centimeters. The parameters studied included VAT and SAT. VAT, representing intra-abdominal omental fat (without ectopic fat), was distinguished from SAT by tracing along the fascial plane defining the internal abdominal wall, and the area was calculated in centimeters. Ectopic liver fat was measured using liver intensity using Osirix software employing the Dixon method (Liver Fat percentage = 100X ((Signal intensity liver/signal intensity spleen) on in-phase T1 (signal intensity liver/signal intensity spleen) on out-phase T1)/2x (signal intensity liver/signal intensity spleen) on in-phase T1).

### Transcriptional profiling of adipose tissue

Adipose tissue biopsies were obtained from a subset of 85 participants from the main cohort. These individuals were selected based on surgical eligibility and consent, and included both MetS and non-MetS cases. From these biopsies, we generated 118 transcriptome datasets:

Visceral and abdominal subcutaneous fat: Paired samples from 33 individuals (23 with MetS, 10 without MetS), yielding 66 datasets.

Peripheral subcutaneous fat: Single depot samples from 52 individuals (36 with MetS, 16 without MetS), yielding 52 datasets.

These datasets were previously submitted to the NCBI GEO database (accession number GSE78721).

### Statistical and bioinformatics analyses

The study employed various statistical methods, including analysis of variance, chi-square, and correlation analysis, all conducted within the Python environment. For sensitivity-specificity analysis, Python libraries numpy, matplotlib, and sklearn were used and confusion matrices were created based on actual and predicted labels. Sensitivity, Specificity, Precision, and F1 score were calculated using following equations.


$$\text{Senstivity}=\frac{\text{True Positive }(TP)}{\text{True Positive }(T\text{P})+\text{False Negative }(FN)}$$

$$\text{Specificity}=\frac{\text{True Negative}(TN)}{\text{True Negative }(TN)+\text{False Positive }(FP)}$$

$$\text{Precision}=\frac{\text{True Positive }(TP)}{\text{True Positive }(TP)+\text{False Positive }(FP)}$$

$$\text{F}1\text{ score}=2\text{ X }\frac{\text{Precision X Senstivity}}{\text{Precision }+\text{ Senstivity}}$$

The F1-score provides a balanced measure of precision and recall, which is crucial for evaluating predictive model performance.

Microarray dataset analysis was conducted using the Bioconductor R packages gcrma and benefiter. Because the annotation package for the prime-view was unavailable, it was generated using the human.db0 and AnnotationForge packages with the prime-view annotation file. To correlate gene expression values with the presence/absence of cutaneous signs, T2D, and MetS, Weighted Gene Correlation Network Analysis (WGCNA) (45) was performed. This method identifies modules of co-expressed genes, assigns colors to these modules for visual representation, and estimates Module Eigengene (ME) to relate them to external traits. WGCNA was performed separately for femoral, visceral, and subcutaneous fat microarray datasets for AC, AN, T2D, and MetS using a soft threshold power β = 15 to ensure scale-free topology. Subsequently, differential gene expression analysis was conducted between the cutaneous-sign positive (CP) and negative (CN) datasets in each depot using limma, with enriched KEGG pathways examined using the WebGeStalt tool (46) for differentially expressed genes (*P* < 0.05). Full DEG lists and KEGG pathway enrichment results are available in **Supplementary Files S1** and **S2**, respectively. Depot-wise counts were as follows: femoral fat (17 CP: 32 CN), subcutaneous (10 CP: 22 CN), and visceral subcutaneous (10 CP: 22 CN).

## Results

### General characteristics

The general clinical, biochemical, and radiological parameters of the participants are shown in **Supplementary Table 1**. Out of 371 individuals, 197 had T2D, 46 had impaired fasting glucose (IFG) and 128 were normal glucose tolerant (NGT). AC, AN and both the signs were present in 27, 50, and 78 Individuals respectively. The number of MetS components, ranging from 0 to 5 were present in 20, 45, 91, 87, 93, and 35, participants respectively. All individuals presenting with AN, AC, or both were clinically diagnosed with T2D, as confirmed by medical records and diagnostic criteria. This context supports the interpretation of elevated fasting glucose levels in these groups as reflective of established diabetes rather than undiagnosed hyperglycemia. Only 8 individuals in our cohort presented with cutaneous signs of insulin resistance despite having normal W:H ratios. This subgroup is indeed small but clinically relevant, as it highlights the occurrence of AN and AC independent of central adiposity.

### Comparison of study parameters between individuals with normal & elevated W:H ratio (CO)

All lipid and glycemic parameters, the number of MetS components, HOMA-IR, HOMA-β, ectopic liver fat, and the prevalence of both cutaneous signs were higher in individuals with an elevated W:H ratio (**Table 1**). However, the visceral and subcutaneous adipose tissue mass was comparable between the two groups.

**Table 1:** Comparison of characteristics between normal and elevated waist-to-hip ratio (W:H ratio) groups using t-test.

Characteristic	Mean (variance) normal W:H	Mean (variance) elevated W:H ratio (CO)	*P* value (T- statistics)
Age (years)	47.24 (217.26)	52.55 (152.93)	**0.0108** (-2.1)
BMI (kg/m^2^)	22.8 (14.9)	24.2 (17.4)	**0.01** (-2.2)
Waist Circumference	80.97(57.92)	88.92(104.36)	**0.0001**(-4.12)
Acanthosis Nigricans %	0.142 (0.12)	0.37 (0.23)	**0.0001** (-3.7)
Acrochordons %	0.14 (0.12)	0.3 (0.214)	**0.005** (-2.6)
Fasting glucose level (mg/dL)	130.1 (4564.49)	157.3 (6083.06)	**0.009** (-2.4)
HbA1c	6.52 (8.03)	7.94 (7.43)	**0.0035(-3.06)**
Insulin (mU/L)	6.9 (58.19)	10.17 (115.3)	**0.008** (-2.4)
Total cholesterol (mg/dL)	156.6 (2353.09)	187.5(2042.3)	**0.0001** (-3.9)
Triglyceride (mg/dL)	128.16 (6096.08)	161.3 (7831.2)	**0.006** (-2.5)
VLDL (mg/dL)	26.3(279.8)	34.5(434.4)	**0.002**(-2.9)
HDL (mg/dL)	42.8 (53.3)	43.8 (72.1)	0.18 (-0.8)
LDL (mg/dL)	87.8 (1537.4)	104.6 (1252.3)	**0.005**(-2.6)
HOMA-β	12.45(872.8)	33.6 (5025.02)	**0.0002** (-3.5)
HOMA-R	1.24 (7.19)	2.7 (32.2)	**0.0019** (-2.9)
Ectopic liver	5.2 (2.2)	7.52 (16.5)	**2.21882E-05** (-4.3)
Subcutaneous fat (cm^2^)	131.5 (1349.5)	141.36(4807.7)	0.1 (-0.98)
Visceral Fat (cm^2^)	145.3(825.6)	141.8(4219.4)	0.3(0.4)
MetS components are present	1.8 (1.6)	2.9 (1.6)	**2.3417727E-06** (-5.1)

W:H ratio of ≥ 0.90 for males and ≥ 0.85 for females was considered indicative of central obesity (CO), based on established South Asian anthropometric standards. Bold values indicate statistically significant results (P < 0.05).

### Comparison of study parameters between individuals without any (non-CSIR) or with any (Ac or AN) or both the cutaneous signs of IR

Significant differences in BMI were observed in ANOVA test among the groups (F = 14.12, *P* < 0.00001) (**Table 2**). Groups with cutaneous signs, especially AN, tend to have higher BMI compared to those without cutaneous signs. Waist circumference shows significant variation among the groups (F = 17.58, *P* < 0.00001), with higher values in groups with cutaneous signs. Similarly, significant differences are observed in W:H ratio across the groups (F = 5.55, *P* = 0.004). Higher ratios are observed in groups with cutaneous signs, particularly those with AC.

**Table 2:** Comparison of clinical characteristics among individuals with different cutaneous signs.

Characteristic	No cutaneous sign (both AC & AN absent) N = 216	AN present, AC absent N= 27	AC present AN absent N=50	Both cutaneous signs present N=78	F-score	*P* value
Age (years)	52.17 ± 13.95	50.32 ± 12.46	53.96 ± 9.35	51.68 ± 10.28	0.70	0.49
BMI (kg/m^2^)	22.94 ± 3.58	25.91 ± 4.60	25.36 ± 5.43	25.77 ± 3.85	14.12	**10^-5^**
Waist circumference	86.91 ± 9.80	95.16 ± 7.80	91.94 ± 7.74	94.88 ± 8.66	17.58	**10^-5^**
Waist-to-hip ratio	0.95 ± 0.08	0.98 ± 0.08	0.99 ± 0.07	0.98 ± 0.09	5.55	**4 X 10^-3^**
Fasting glucose level	123.31 ± 58.76	193.7 ± 63.10	187.43 ± 75.85	203.12 ± 89.88	35.30	**10^-5^**
Hb1Ac (%)	6.68 ± 2.49	9.02 ± 1.77	8.88 ± 2.61	9.48 ± 2.87	25.82	**10^-5^**
Total cholesterol	176.69 ± 44.48	187.01 ± 41.0	200.89 ± 47.33	196.74 ± 51.43	4.22	**10^-2^**
Triglyceride	145.55 ± 88.58	168.78 ± 89.94	188.87 ± 93.49	173.09 ± 77.81	3.70	**2 X 10^-2^**
HDL (mg/dL)	43.91 ± 7.90	43.67 ± 7.64	42.90 ± 6.48	43.71 ± 10.50	0.20	0.81
Insulin (IU/L)	7.02 ± 9.68	13.64 ± 15.84	8.23 ± 7.94	11.68 ± 10.58	5.23	**0.0012**
HOMA-β	111.85 ± 235.62	34.15 ± 37.10	66.05 ± 96.17	40.25 ± 47.46	3.18	**4 X 10^-2^**
HOMA-R	2.64 ± 3.41	4.65 ± 3.59	8.0 ± 10.79	6.21 ± 8.53	18.19	**10^-5^**
Visceral Fat	135.09 ± 60.62	174.85 ± 57.16	113.13 ± 55.95	151.07 ± 57.60	4.46	**10^-2^**
Subcutaneous Fat	131.80 ± 47.69	150.96 ± 69.97	132.87 ± 99.18	172.10 ± 110.55	1.09	0.34
Ectopic Liver Fa	6.27 ± 3.05	9.40 ± 3.56	12.15 ± 10.35	7.75 ± 2.74	10.77	**5 X 10^-5^**
HDL (mg/dL)	43.91 ± 7.90	43.67 ± 7.64	42.90 ± 6.48	43.71 ± 10.50	0.20	0.81
Average number. of MetS Components present	2.26 ± 1.30	3.48 ± 0.9	3.81 ± 1.14	3.46 ± 1.0	33.98	**10^-5^**

Bold values indicate statistically significant results (P < 0.05).

A substantial difference in fasting glucose levels is noted among the groups (F = 35.30, *P* < 0.00001). Groups with cutaneous signs, especially those with both AN and AC, have markedly higher glucose levels, suggesting a correlation between the presence of these signs and hyperglycemia. Furthermore, significant differences in HbA1c levels are seen among the groups (F = 25.82, *P* < 0.00001). Higher HbA1c in groups with cutaneous signs suggests that these groups are more likely to have poorly controlled diabetes.

The differences in total cholesterol levels are statistically significant (F = 4.22, *P* = 0.04). Higher cholesterol levels are associated with the presence of AC, indicating potential lipid metabolism issues in these groups. There are significant differences in triglyceride levels among the groups (F = 3.70, *P* = 0.02). Elevated triglycerides are more common in groups with cutaneous signs, suggesting dyslipidemia.

Significant differences in insulin levels are observed (F = 5.23, *P* = 0.0012), with the highest levels in groups with AN. This indicates insulin resistance, especially in those with AN. Differences in HOMA-β values are significant (F = 3.18, *P* = 0.04). Lower HOMA-β values in groups with AN suggest impaired β-cell function. Significant differences in HOMA-R values are seen (F = 18.19, *P* < 0.00001), with the highest values in groups with cutaneous signs, reflecting insulin resistance.

Visceral fat differs significantly among the groups (F = 4.46, *P* = 0.04). Higher visceral fat is observed in groups with AN, indicating a link with central obesity. No significant differences in subcutaneous fat are found (F = 1.09, *P* = 0.34), suggesting that subcutaneous fat is not related to the presence of cutaneous signs.

Ectopic liver fat shows significant differences among the groups (F = 10.77, *P* < 0.00005). Higher liver fat content is noted in groups with cutaneous signs, especially AC, indicating hepatic steatosis.

There are significant differences in the average number of “MetS clinical” components (F = 33.98, *P* < 0.00001). Groups with cutaneous signs, particularly both AN and AC, tend to have more MetS components, highlighting their role as bedside markers for metabolic syndrome.

The findings suggest that groups with cutaneous signs, particularly those with both AN and AC, have significantly worse metabolic profiles, including higher BMI, waist circumference, glucose levels, insulin resistance, and more components of metabolic syndrome. These results highlight a strong association between cutaneous signs like AN and AC and conditions such as metabolic syndrome, obesity, and type 2 diabetes.

### Comparison of study parameters between individuals with any cutaneous signs of IR with central obesity as compared with those with central obesity or with neither central obesity nor cutaneous signs of IR

BMI, Waist Circumference, fasting glucose, HbA1c, LDL, cholesterol, insulin, HOMA-R, HOMA-B, liver fat, MetS components had very low *P*-values (all < 0.001), indicating statistically significant differences across the groups (**Table 3**). The high ANOVA F-statistics for fasting glucose: 51.5, and HbA1c: 55.5 suggested that the variance between the groups was much greater than the variance within the groups, reinforcing that the differences were highly significant.

**Table 3:** Comparison of clinical characteristics among individuals belonging to either Normal W:H + No Cutaneous sign (Non-CO-CSIR), Elevated W:H + No Cutaneous sign (CO), and Elevated W:H + Any Cutaneous sign (CO-CSIR).

S. No.	Feature	F-statistic	P-value
1	BMI	22.9	**4.45 × 10^−10^**
	Waist circumference	33.5	**3.45 × 10^−19^**
2	Fasting Glucose	51.5	**2.09 × 10^−20^**
3	Hb1Ac	55.5	**9.86 × 10^−22^**
4	VLDL	3.4	**3.39 × 10^−2^**
5	LDL	13.0	**3.67 × 10^−6^**
	HDL	0.53	0.66
6	Triglycerides	6.5	**1.68 × 10^−3^**
7	Cholesterol	14.6	**7.96 × 10^−7^**
8	Insulin	5.1	**6.79 × 10^−3^**
9	HOMA-R	16.7	**1.14 × 10^−7^**
10	HOMA- β	9.0	**1.51 × 10^−4^**
11	Visceral fat	1.67	0.18
12	Subcutaneous fat	1.73	0.16
13	Liver fat	9.6	**1.15 × 10^−4^**
14	MetS component	61.5	**1.06 × 10^−23^**

Bold values indicate statistically significant results (P < 0.05).

Further VLDL, triglycerides, insulin also shown statistically significant differences among the groups with moderate F-statistics and *P*-values just below 0.05.

The *P*-values and F-statistics for HDL, visceral fat, and subcutaneous fat indicated no statistically significant differences among the groups.

ANOVA therefore indicates that there is a difference among the group means; however, to determine specifically which groups differ from each other among them, Tukey’s HSD (Honestly Significant Difference) *post hoc* tests were performed to identify those specific group differences (**Table 4**).

**Table 4:** Results of Tukey’s HSD *post hoc* analysis for the ANOVA results obtained by comparing clinical characteristics among individuals in three groups: Normal W:H + No Cutaneous sign (Non-CO-CSIR), Elevated W:H + No Cutaneous sign (CO), and Elevated W:H + Any Cutaneous sign (CO-CSIR).

S. No.	Characteristics	Group1	Group2	Mean difference	P-adjusted
1	BMI	Non-CO-CSIR	CO-CSIR	3.6	**0.0000**
		CO	CO-CSIR	2.6	**0.0000**
2	Waist Circumference	Non-CO-CSIR	CO-CSIR	0.7	**0.0000**
		CO	CO-CSIR	0.3	**0.0034**
3	Acanthosis Nigricans	Non-CO-CSIR	CO-CSIR	0.8	**0.0000**
		CO	CO-CSIR	0.8	**0.0000**
4	Acrocordons	Non-CO-CSIR	CO-CSIR	0.7	**0.0000**
		CO	CO-CSIR	0.7	**0.0000**
5	Fasting Glucose	Non-CO-CSIR	CO-CSIR	84.9	**0.0000**
		CO	CO-CSIR	71.3	**0.0000**
6	Hb1Ac	Non-CO-CSIR	CO-CSIR	3.2	**0.0000**
		CO	CO-CSIR	2.6	**0.0000**
7	Cholesterol	Non-CO-CSIR	CO-CSIR	45.5	**0.0000**
		CO	CO-CSIR	12.8	**0.0283**
	HDL	CO	CO-CSIR	1.1	0.05
		Non-CO-CSIR	CO-CSIR	1.7	0.05
8	LDL	Non-CO-CSIR	CO-CSIR	31.3	**0.0000**
		CO	CO-CSIR	12.9	**0.0027**
9	Triglycerides	Non-CO-CSIR	CO-CSIR	47.9	**0.0112**
		CO	CO-CSIR	28.6	**0.0089**
10	Insulin	Non-CO-CSIR	CO-CSIR	5.2	**0.0226**
		CO	CO-CSIR	2.9	**0.0328**
11	HOMA-R	Non-CO-CSIR	CO-CSIR	4.2	**0.0003**
		CO	CO-CSIR	3.4	**0.0000**
12	HOMA-B	Non-CO-CSIR	CO-CSIR	-138.6	**0.0003**
		CO	CO-CSIR	-55.9	**0.0178**
13	Visceral fat	Non-CO-CSIR	CO-CSIR	11.0	0.56
		CO	CO-CSIR	12.6	0.64
14	Subcutaneous fat	Non-CO-CSIR	CO-CSIR	15.3	0.62
		CO	CO-CSIR	10.2	0.69
15	Liver fat	Non-CO-CSIR	CO-CSIR	3.3	**0.0004**
		CO	CO-CSIR	2.1	**0.0017**
16	MetS	Non-CO-CSIR	CO-CSIR	2.0	**0.0000**
		CO	CO-CSIR	1.2	**0.0000**
17	T2D	Non-CO-CSIR	CO-CSIR	0.8	**0.0000**
		CO	CO-CSIR	0.6	**0.0000**

Bold values indicate statistically significant results (P < 0.05).

There is a significant mean difference of 3.6 between Non-CO-CSIR and CO-CSIR (F = 22.9, *P* = 0.0) for BMI, indicating that individuals with elevated W: H ratio and cutaneous signs have significantly higher BMI compared to those with normal W:H ratio and no cutaneous signs. A significant mean difference of 2.6 was observed between CO and CO-CSIR (F = 22.9, *P* = 0.0), suggesting that the presence of cutaneous signs in individuals with elevated W:H ratio further increases BMI.

The mean difference in waist circumference is 0.7 between Non-CO-CSIR and CO-CSIR (F = 33.5, *P* = 0.0), indicating that waist circumference is higher in individuals with elevated W:H ratio and cutaneous signs. A smaller but significant difference of 0.3 (F = 33.5, *P* = 0.0034) was found between CO and CO-CSIR, suggesting that cutaneous signs also associated with higher waist circumference among those with elevated W:H ratio.

Significant differences were found in the prevalence of AN and AC with a mean difference of 0.8 for AN and 0.7 for AC between Non-CO-CSIR and CO-CSIR, and CO and CO-CSIR, all with highly significant values (F = varies, *P* = 0.0). This indicates that prevalence of these cutaneous signs are much more higher in individuals with both normal and elevated W:H ratio.

Substantial differences in fasting glucose (84.9 and 71.3) and HbA1c levels (3.2 and 2.6) were observed between Non-CO-CSIR and CO-CSIR, and CO and CO-CSIR, respectively (F = 51.5 for Glucose, F = 55.5 for HbA1c, *P* = 0.0). This shows a significant increase in these markers of glycemic control as individuals transition from a normal W: H ratio without cutaneous signs to an elevated W:H ratio with cutaneous signs.

Significant differences in cholesterol, LDL, and triglycerides levels were observed between the groups, particularly between Non-CO-CSIR and CO-CSIR, with F = 14.6 for Cholesterol, F = 13.0 for LDL, and F = 6.5 for Triglycerides, *P* < 0.05. This suggests that lipid abnormalities are more pronounced in individuals with elevated W:H ratio and cutaneous signs.

Insulin levels and insulin resistance (HOMA-R) showed significant increases with positive mean differences between Non-CO-CSIR and CO-CSIR, and CO and CO-CSIR (F = 5.1 for Insulin, F = 16.7 for HOMA-R, *P* < 0.05). However, HOMA- β (which reflects β-cell function) showed a significant decrease, particularly between Non-CO-CSIR and CO-CSIR (F = 9.0, *P* < 0.05), implying worsening β-cell function with the addition of cutaneous signs.

There is a significant increase in liver fat content between the groups, with the highest values in the CO-CSIR group (F = 9.6, *P* = 0.0004), indicating a greater risk of hepatic steatosis associated with both elevated W:H ratio and the presence of cutaneous signs.

The analysis reveals highly significant differences in the prevalence of number of MetS components and T2D between Non-CO-CSIR and CO-CSIR, and CO and CO-CSIR (F = 61.5 for MetS, F = varies for T2D, *P* = 0.0000). This suggests that both elevated W:H ratio and the presence of cutaneous signs significantly increase the risk of developing MetS and T2D.

The BMI is significantly higher in the CO-CSIR group compared to both the Non-CO-CSIR group (mean difference = 3.6, *P*-adjusted = 0.0000) and the CO group (mean difference = 2.6, *P*-adjusted = 0.0). Waist circumference is significantly greater in the CO-CSIR group compared to the Non-CO-CSIR group (mean difference = 0.7, *P*-adjusted = 0.0) and the CO group (mean difference = 0.3, *P*-adjusted = 0.0034).

The prevalence of AN is significantly higher in the CO-CSIR group compared to both the Non-CO-CSIR group (mean difference = 0.8, *P*-adjusted = 0.0) and the CO group (mean difference = 0.8, *P*-adjusted = 0.0). The prevalence of AC is significantly higher in the CO-CSIR group compared to both the Non-CO-CSIR group (mean difference = 0.7, *P*-adjusted = 0.0) and the CO group (mean difference = 0.7, *P*-adjusted = 0.0).

Fasting glucose levels are significantly higher in the CO-CSIR group compared to both the Non-CO-CSIR group (mean difference = 84.9, *P*-adjusted = 0.0) and the CO group (mean difference = 71.3, *P*-adjusted = 0.0). HbA1c levels are significantly higher in the CO-CSIR group compared to both the Non-CO-CSIR group (mean difference = 3.2, *P*-adjusted = 0.0) and the CO group (mean difference = 2.6, *P*-adjusted = 0.0).

Cholesterol levels are significantly higher in the CO-CSIR group compared to both the Non-CO-CSIR group (mean difference = 45.5, *P*-adjusted = 0.0) and the CO group (mean difference = 12.8, *P*-adjusted = 0.0283). HDL levels are borderline significantly lower in the CO-CSIR group compared to both the Non-CO-CSIR group (mean difference = 1.7, *P*-adjusted = 0.05) and the CO group (mean difference = 1.1, *P*-adjusted = 0.05). LDL levels are significantly higher in the CO-CSIR group compared to both the Non-CO-CSIR group (mean difference = 31.3, *P*-adjusted = 0.0) and the CO group (mean difference = 12.9, *P*-adjusted = 0.0027). Triglyceride levels are significantly higher in the CO-CSIR group compared to both the Non-CO-CSIR group (mean difference = 47.9, p-adjusted = 0.0112) and the CO group (mean difference = 28.6, *P*-adjusted = 0.0089).

Insulin levels are significantly higher in the CO-CSIR group compared to both the Non-CO-CSIR group (mean difference = 5.2, *P*-adjusted = 0.0226) and the CO group (mean difference = 2.9, *P*-adjusted = 0.0328). HOMA-R is significantly higher in the CO-CSIR group compared to both the Non-CO-CSIR group (mean difference = 4.2, *P*-adjusted = 0.0003) and the CO group (mean difference = 3.4, *P*-adjusted = 0.0). HOMA-B is significantly lower in the CO-CSIR group compared to both the Non-CO-CSIR group (mean difference = -138.6, *P*-adjusted = 0.0003) and the CO group (mean difference = -55.9, *P*-adjusted = 0.0178). There is no significant difference in visceral fat between the Non-CO-CSIR and CO-CSIR groups (mean difference = 11.0, *P*-adjusted = 0.56) or between the CO and CO-CSIR groups (mean difference = 12.6, *P*-adjusted = 0.64). Similarly, there is no significant difference in subcutaneous fat between the Non-CO-CSIR and CO-CSIR groups (mean difference = 15.3, *P*-adjusted = 0.62) or between the CO and CO-CSIR groups (mean difference = 10.2, *P*-adjusted = 0.69).

Liver fat is significantly higher in the CO-CSIR group compared to both the Non-CO-CSIR group (mean difference = 3.3, *P*-adjusted = 0.0004) and the CO group (mean difference = 2.1, *P*-adjusted = 0.0017).

The prevalence of MetS components is significantly higher in the CO-CSIR group compared to both the Non-CO-CSIR group (mean difference = 2.0, *P*-adjusted = 0.0) and the CO group (mean difference = 1.2, *P*-adjusted = 0.0).

The prevalence of T2D is significantly higher in the CO-CSIR group compared to both the Non-CO-CSIR group (mean difference = 0.8, *P*-adjusted = 0.0) and the CO group (mean difference = 0.6, *P*-adjusted = 0.0).

### Sensitivity–specificity analysis

We conducted sensitivity-specificity analyses across various anthropometric, glycemic, and cutaneous phenotypes to determine their association with “MetS clinical”. (**Table 5**).

**Table 5:** Performance matrices for evaluation of various anthropometric parameters, cutaneous signs, and HOMA-IR for prediction of MetS status.

Evaluation metric	BMI (> 27.5)	BMI (> 30)	CSIR (AC/AN)	Waist circumference	W:H Ratio (CO)	CO-CSIR
Sensitivity (True Positive Rate)	0.27	0.11	0.61	0.87	**0.94**	0.63
Specificity (True Negative Rate)	0.93	**0.98**	0.85	0.65	0.19	0.84
Precision	0.84	**0.89**	0.85	0.78	0.62	0.86
F1 Score	0.41	0.20	0.71	**0.82**	0.75	0.73

Bold values indicate statistically significant results (P < 0.05).

BMI is not a complete measure of metabolic health. Our analysis revealed that BMI > 27.5 kg/m^2^ or even > 30 kg/m^2^ corresponded to only 27% and 11% sensitivity, respectively, in predicting MetS status. Despite, an elevated waist circumference (> 80 cm in females and > 90 cm in males) is a component of MetS clinical, we found that an elevated W:H ratio (> 0.85 in females and > 0.90 in males) serves as a better denominator of MetS, with 94% sensitivity in predicting MetS clinical.

CO-CSIR strikes a balance between sensitivity, specificity, and precision, making it a more robust predictor of MetS clinical status. W:H ratio (CO) has the highest sensitivity and a relatively good F1 score but fails in specificity and precision, indicating that it might lead to more false positives. CSIR alone is less effective than CO-CSIR in terms of both sensitivity and F1 score, though it has marginally higher specificity. Overall, CO-CSIR provides a more balanced and accurate prediction of MetS status compared to either CO or CSIR alone, making it the better predictor.

**Figure 1:**
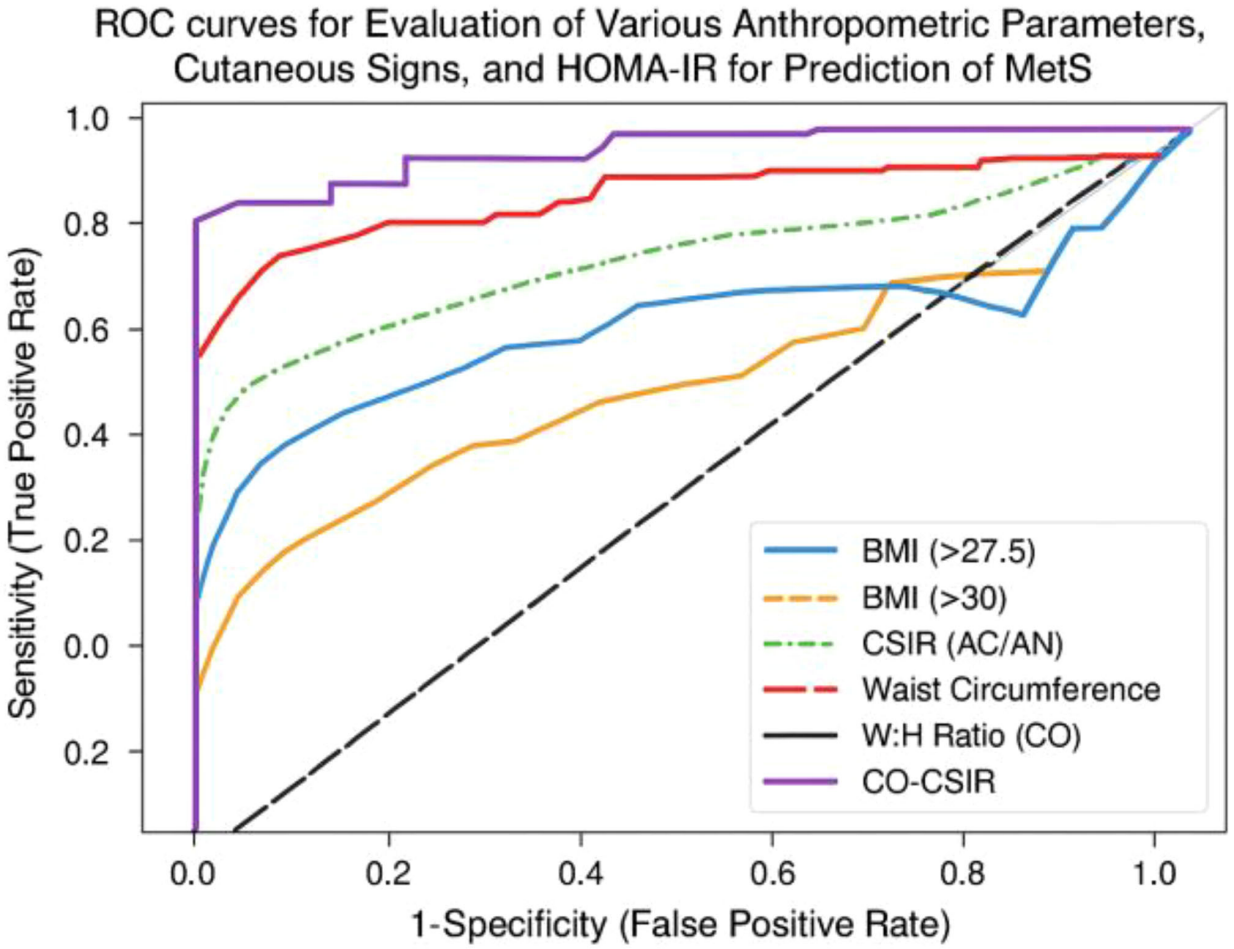
ROC plot comparing the predictive performance of BMI, CSIR, waist circumference, W:H ratio, and CO-CSIR for MetS clinical status.

To further visualize the diagnostic performance of these markers, we generated ROC-curves comparing BMI thresholds, CSIR, waist circumference, W:H ratio, and CO-CSIR. The ROC plot illustrates the trade-off between sensitivity and false positive rate (1 – specificity) across these predictors (**Figure 1**). CO-CSIR demonstrated the most balanced curve, with high sensitivity and specificity, outperforming individual markers in overall diagnostic accuracy.

The **Table 6** provides a comprehensive overview of the performance of different metrics—W: H ratio (CO), CSIR, and CO-CSIR—in predicting MetS status, based on the number of MetS components (from 5 to ≥1).

**Table 6:** Performance matrices for evaluation of W:H ratio (CO), Cutaneous Signs of Insulin Resistance (CSIR), and CO-CSIR in predicting number of components of MetS (highest values is marked bold).

Evaluation Metric	Number of MetS Components				
	5	≥ 4	≥ 3	≥ 2	≥ 1
	W:H ratio (CO)				
Sensitivity (True Positive Rate)	**0.97**	0.97	0.94	0.92	0.90
Specificity (True Negative Rate)	0.12	0.16	0.19	0.25	**0.40**
Precision	0.1	0.38	0.62	0.85	**0.96**
F1 Score	0.19	0.54	0.75	0.88	**0.93**
Cutaneous Signs of Insulin Resistance (CSIR)					
Sensitivity (True Positive Rate)	**0.74**	0.66	061	0.49	0.42
Specificity (True Negative Rate)	0.62	0.71	0.85	0.92	**1.00**
Precision	0.17	0.54	0.85	0.97	**1.00**
F1 Score	0.27	0.59	**0.71**	0.65	0.59
CO-CSIR					
Sensitivity (True Positive Rate)	**0.71**	0.64	0.59	0.47	0.42
Specificity (True Negative Rate)	0.64	0.73	0.87	0.94	**1.0**
Precision	0.17	0.56	0.86	0.97	**1.0**
F1 Score	0.27	0.60	**0.70**	0.63	0.59
CO-Non-CSIR					
Sensitivity (True Positive Rate)	1.0	**0.95**	0.92	0.88	0.87
Specificity (True Negative Rate)	0.16	0.19	0.20	0.25	**0.40**
Precision	005	0.23	0.42	0.75	**0.93**
F1 Score	0.09	0.37	0.57	0.81	**0.90**

W: H Ratio excels in sensitivity, making it a strong predictor for detecting the presence of MetS, especially when multiple components are present. However, its low specificity indicates a tendency for false positives. CSIR and CO-CSIR have more balanced performances, with higher specificity and precision. CO-CSIR offers a slight edge in specificity compared to CSIR, making it a reliable metric for identifying true negatives and avoiding over-diagnosis. We could therefore conclude that while the CO is effective in identifying MetS cases due to its high sensitivity, the combination of CO-CSIR provides a more balanced approach, particularly in clinical settings where specificity and precision are crucial for accurate diagnosis.

CO-CSIR offers a comprehensive evaluation by combining central obesity and insulin resistance markers, resulting in a well-rounded predictive tool for MetS. In other words, the CO-CSIR is the most sensitive bedside predictor of MetS. Despite its moderate specificity, individuals with these bedside signs exhibited higher insulin resistance, triglyceride levels, and glucose levels, indicating dysmetabolism.

### WGCNA analysis

**Figure 2:**
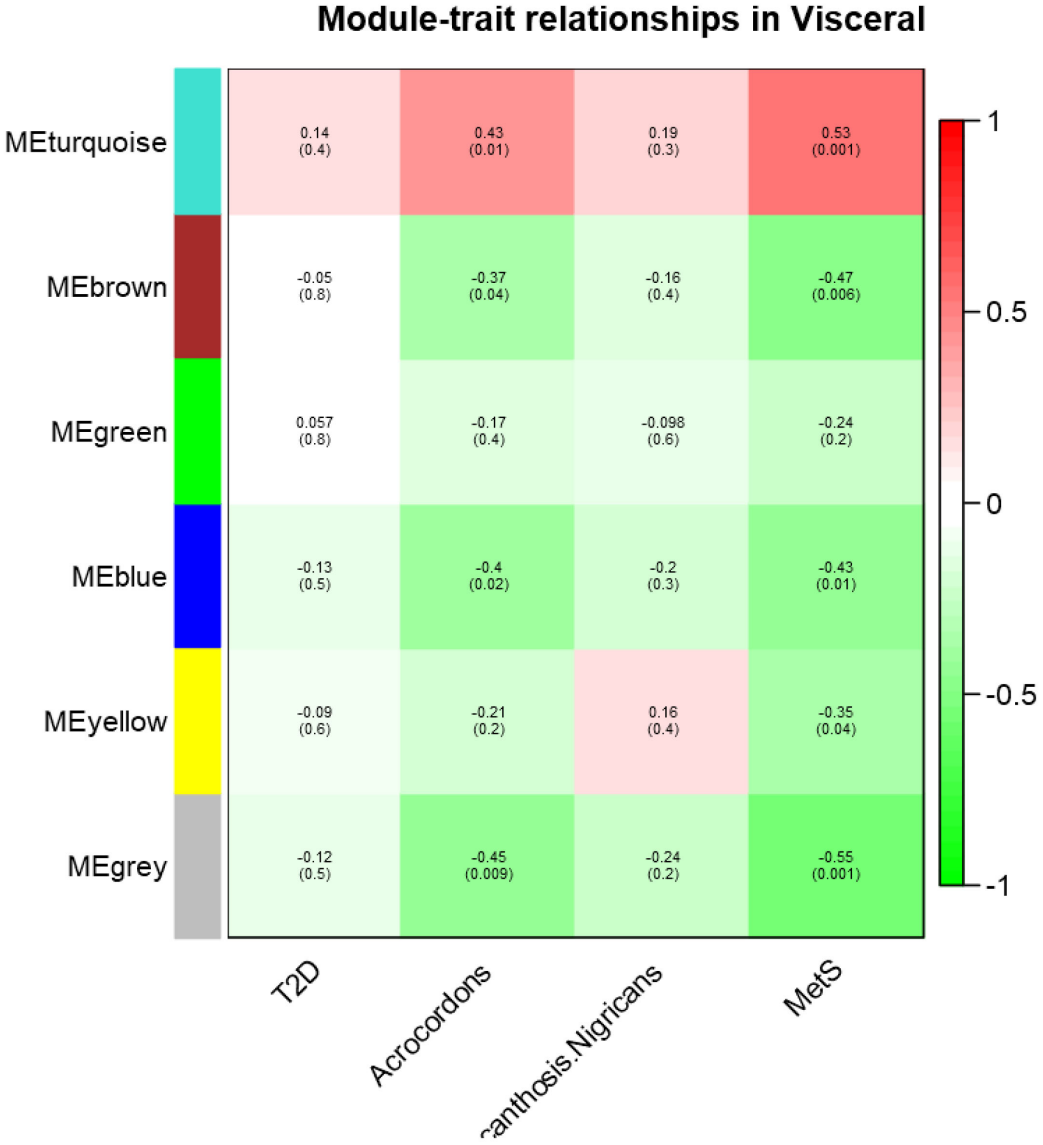
Module- trait relationship in visceral datasets for T2D, MetS, Acrochordon, and Acanthosis Nigricans.

In visceral fat, three modules (brown, blue, and gray) showed a significant negative correlation, while one module (twilight) exhibited a positive correlation with AC and MetS clinical (as shown in **Figure 2**).

The directionality of correlation in the four modules indicates an association between these traits. We therefore posit that gene expression in visceral fat is indeed the probable denominator of MetS clinical and that the occurrence of skin tags is somehow linked to clinical diseases. We also conducted pathway enrichment analysis for these statistically significant modules in visceral depot, which is presented in the **Supplementary File**. Some of the relevant pathways enriched in these modules could indicate hallmark derangements of MetS clinical, such as low-grade inflammation, oxidative and endoplasmic reticular (ER) stress, and mitochondrial dysfunction (Refer to **Supplementary File** for a module-wise list of enriched pathways).

The IL-18, white fat cell differentiation, chemokine, and IL-11 signaling pathways enriched in these modules contribute to low-grade inflammation through various mechanisms. For instance, IL-18, a pro-inflammatory cytokine, enhances the production of other pro-inflammatory cytokines like IFN-γ and activates immune cells, leading to sustained inflammation via the NF-κB and MAPK pathways (47). In white adipose tissue, expansion associated with obesity results in the secretion of adipokine such as TNF-α and IL-6, which recruit and activate macrophages, perpetuating inflammation (48). Chemokines direct immune cell migration to inflammatory sites, and chronic production of chemokine like CCL2 leads to continuous immune cell infiltration while maintaining a low-grade inflammatory state (49). IL-11, which is traditionally considered anti-inflammatory, can also contribute to inflammation by activating the JAK/STAT3 pathway, promoting pro-inflammatory gene expression, and fibrosis when chronically activated (50). The pathways regulating Hippo signaling and oxidative phosphorylation contribute to oxidative and ER stress through their roles in cellular homeostasis and energy metabolism. Dysregulation of the Hippo signaling pathway can impair cell proliferation and apoptosis, leading to cellular stress and promoting reactive oxygen species (ROS) production, which induces oxidative stress (51). In oxidative phosphorylation, mitochondrial dysfunction can result in inefficient electron transport and increased ROS production, directly causing oxidative damage and subsequent ER stress due to the accumulation of misfolded proteins in the ER (48). The electron transport chain (ETC) within the OXPHOS system is essential for ATP production, but its dysfunction can lead to excessive ROS generation, which damages mitochondrial components and impairs function (52). Exercise-induced circadian regulation affects mitochondrial dynamics and bioenergetics; misalignment of circadian rhythms with exercise timing can disrupt mitochondrial function and energy production (53). **Table 7** provides a structured summary of the key findings of WGCNA.

**Table 7:** Key WGCNA modules associated with cutaneous signs and metabolic traits.

Depot	Module color	Trait correlation	Top enriched pathways (KEGG)	Representative hub genes	Biological relevance
Visceral Fat	Blue	AC, MetS	Adipocytokine signaling, TNF signaling	*LEP*, *IL6*, *SOCS3*	Inflammation, adipose dysfunction
	Turquoise	AN, T2D	Insulin signaling, PI3K-Akt pathway	*IRS1*, *AKT2*, *FOXO1*	Insulin resistance, glucose metabolism
Subcutaneous Fat	Brown	AC	ECM-receptor interaction, focal adhesion	*COL6A3*, *FN1*, *ITGA5*	Tissue remodeling, adipose expansion
Femoral Fat	Yellow	MetS	PPAR signaling, fatty acid metabolism	*PPARG*, *FABP4*, *ADIPOQ*	Lipid metabolism, adipocyte differentiation

## Discussion

The cutaneous signs of IR and the anthropometric measures of obesity, particularly those assessing central obesity, are well-known for their association with high IR, T2D and other clustering diseases. However, from a clinical practice perspective, the most pertinent question is whether these physical signs can be used for bedside prediction of MetS, both as a cluster of diseases and the underlying adipocentric metabolic dysfunction, particularly in South Asians who exhibit the thin fat phenotype. The findings of the present study support the view that the presence of any cutaneous sign of IR in individuals with a high W:H ratio (CO-CSIR) is a very useful physical sign of MetS (MetS clinical). Presence of W:H ratio above the cut-off values of 0.85 and 0.90 respectively in women and men is sensitive sign of “MetS clinical” and on the top of that presence of any cutaneous sign of IR is a very specific sign of the same. Interestingly, CO-CSIR individuals had high IR, impaired beta-cell function, dyslipidemia, hyperglycemia, ectopic liver fat and a higher prevalence of T2D and other MetS clustering diseases. Moreover, the modules of co-expressed genes in visceral adipose tissue show an association with MetS clinical and acrochordons (AC), and they enriched the pathways of inflammation, oxidative stress, and ER stress. In other words, CO-CSIR could also serve as a physical sign of the “MetS disease or adiposopathy”.

Several previous studies from India have investigated the association between these cutaneous signs of IR in isolation with different facets of MetS like anthropometric measures of obesity (i.e., BMI (37), waist circumference (42, 43), W:H ratio, HOMA-IR (31–40), lipid parameters, diabetes, and other diseases clustering with MetS (39–41). They all found an association between these cutaneous signs and the facets of MetS they investigated. To the best of our knowledge, no previous study has compared both cutaneous signs together for their associations with HOMA-IR and MetS. We observed here that AC, as compared with AN, was associated with a higher degree of IR and more ectopic fat. Therefore, these observations suggest that AC indicates a more severe degree of the underlying adipocentric pathophysiological metabolic dysfunction of MetS. Moreover, as mentioned previously, AC, not AN, showed an association with both MetS and the molecular qualitative traits of adipose tissue pathology in visceral adipose tissue.

To the best of our knowledge, the concept of CO-CSIR, the bedside physical sign of MetS, and adipo-centric dysmetabolism (otherwise so-called adiposopathy) presented in this study, has not been reported in any previous study in South Asia. Whether these two clinical signs, i.e., W:H ratio and AC/AN) represent two different facets of the pathophysiological mechanism of MetS disease or something else cannot be answered by this cross-sectional study. However, the results of the present study support the notion that the presence of cutaneous signs of IR in individuals with central obesity is associated with a higher degree of dysfunction in several facets of adipose tissue pathology-driven mechanisms of MetS disease, i.e., ectopic fat, HOMA-IR, beta-cell dysfunction, hyperglycemia, etc., as well as a higher degree of W:H ratio itself. In other words, CO-CSIR reflects a higher degree of MetS disease as compared with an increased W:H ratio alone. The other facet of the coin is that while keeping apart these potential adipose-centric pathophysiological mechanisms of MetS disease, what is more important clinically is that both physical signs can be easily measured at bedside and in general practice.

The CO group emerges as a critical intermediate phenotype in our analysis. While these individuals exhibit worse metabolic parameters than the Non-CO-CSIR group, they are consistently better than the CO-CSIR group across glycemic, lipid, and insulin resistance indices. This gradient suggests a potential disease spectrum, where central obesity alone may represent an earlier stage of adipocentric dysfunction, and the emergence of cutaneous signs may mark progression toward overt metabolic syndrome. Whether the CO group represents MHO or a preclinical stage of adiposopathy remains an open question. Importantly, their prognosis is uncertain—some may remain stable, while others may transition toward CO-CSIR status. This ambiguity underscores the need for longitudinal studies to track progression and identify early biomarkers of transition. We propose that CO individuals should be considered a high-risk surveillance group, warranting closer clinical monitoring and lifestyle intervention, even in the absence of cutaneous signs.

MetS (ASCVD risk predictor) has been a subject of controversy, both in terms of its utility as a CV risk predictor and the precise cut-off values of the parameters of its clustering diseases and anthropometric measurements in its diagnosis (26–28). Second, despite extensive research in this field, MetS remains distant from being a diagnostic entity in common prescriptions (29–32). We have presented here the concept of a “physical sign”, i.e., CO-CSIR, as a bedside sign for the recognition of not only the cluster of diseases that otherwise constitute MetS but also the adipocentric pathophysiologic mechanisms of MetS like HOMA-IR, ectopic fat, dyslipidemia, impaired beta-cell function, adipose tissue molecular pathology (i.e., MetS disease or adiposopathy). Considering the concept that MetS disease or adiposopathy as a complex genetic disease with clustering diseases (i.e., MetS clinical) as its clinical manifestation, the findings of the present study suggest that CO-CSIR could serve as a useful bedside physical indicator of this disease. However, an important question remains unanswered: Presence of CO without CSIR represent an early stage in its natural history or an incompletely developed MetS disease or adiposopathy? This question cannot be answered in this cross-sectional study. Therefore, longitudinal studies are needed to understand the prognostic clinical importance of these physical signs.

### Limitation

There were several limitations in this study: it was a cross-sectional, single-center and hospital-based study. Given the fact that MetS is not routinely diagnosed in conventional clinical practice, recruiting patients explicitly categorized as having MetS was not feasible. Therefore, in the present study, at time of recruitment, participants were classified as diabetic, IFG or non-diabetic. Moreover, only adults with mean age 51.9 **±** 12.7 were included in this study.

Furthermore, information on the duration of pharmacological treatment was not uniformly available and was therefore not included in the analysis. Future studies should consider treatment duration when evaluating metabolic outcomes.

A key limitation is the inability to assess disease progression. The CO group may represent a transitional phenotype—either metabolically healthy or at an early stage of adipocentric dysfunction. Their prognosis remains uncertain and warrants longitudinal tracking. Therefore, community based large-scale prospective studies are needed to strengthen the concept of CO-CSIR. Moreover, molecular insights into the shared mechanisms and pathways between these physical signs and clustering diseases would be of paramount importance in establishing CO-CSIR as a physical sign of MetS (CV risk predictor) as well as adipose dysfunction-driven dysmetabolism (so-called MetS disease or adiposopathy).

## Conclusion

The presence of raised W:H ratio is a sensitive and on the top of that presence of cutaneous signs of IR (CO-CSIR) is a specific physical sign of metabolic syndrome (MetS clinical) and its underlying pathophysiological pathways and mechanisms (MetS disease or adiposopathy) in Asian Indians.

## Data Availability

The dataset presented in this study may be requested from the corresponding author.
