# Supplementary material for: Cutaneous signs of insulin resistance with central obesity: insights into adipocentric metabolic dysfunction in South Asians
Source: Front Clin Diabetes Healthc. 2025 Dec 3;6:1691675. doi: 10.3389/fcdhc.2025.1691675 (PMC12708322; doi:10.3389/fcdhc.2025.1691675)
Supplement: Supplementary file 2 [file Table2.docx]

**Depot-wise Pathway Enrichment Analysis of Differentially Expressed Genes (DEGs) Between Cutaneous Sign Positive and Negative Adipose Tissue Samples**

**Peripheral Fat**

| **S. No.** | **Gene Set** | **Description** | **Size** | **Expect** | **Ratio** | **P Value** |
| --- | --- | --- | --- | --- | --- | --- |
| 1 | hsa03008 | Ribosome biogenesis in eukaryotes | 81 | 3.8322 | 4.1751 | 8.9795e-7 |
| 2 | hsa03040 | Spliceosome | 133 | 6.2924 | 2.3838 | 0.0014692 |
| 3 | hsa00760 | Nicotinate and nicotinamide metabolism | 30 | 1.4193 | 4.2273 | 0.0024271 |
| 4 | hsa05130 | Pathogenic Escherichia coli infection | 55 | 2.6021 | 3.0744 | 0.0040158 |
| 5 | hsa05205 | Proteoglycans in cancer | 198 | 9.3676 | 1.9215 | 0.0057495 |
| 6 | hsa05212 | Pancreatic cancer | 75 | 3.5483 | 2.5364 | 0.0085144 |
| 7 | hsa04666 | Fc gamma R-mediated phagocytosis | 91 | 4.3053 | 2.3227 | 0.010459 |
| 8 | hsa05131 | Shigellosis | 65 | 3.0752 | 2.6014 | 0.011067 |
| 9 | hsa05230 | Central carbon metabolism in cancer | 65 | 3.0752 | 2.6014 | 0.011067 |
| 10 | hsa01521 | EGFR tyrosine kinase inhibitor resistance | 79 | 3.7376 | 2.4080 | 0.011836 |

Several infections, and immune system related pathways - *Pathogenic Escherichia coli infection, Proteoglycans in cancer, Pancreatic cancer,* and *Shigellosis* were found enriched highlighting the association of skin tags with metabolic derangement. We attempted to assess the link between these pathways with metabolic conditions.

Pathway ‘*Nicotinate and nicotinamide metabolism*’ is relevant to metabolic syndrome. During cellular metabolism and DM, nicotinamide limits insulin resistance and glucose release with additional pathways to prevent the onset and progression of DM. In animal models, nicotinamide can maintain normal fasting blood glucose with streptozotocin-induced. In addition, nicotinamide can markedly improve glucose utilization. Oral nicotinamide administration at a dose of 1200mg/m2/day protects pancreatic β-cell function and prevents clinical disease in islet-cell antibody-positive first-degree relatives of type-1 DM. Patients with recent onset type-1 DM receiving nicotinamide (25mg/kg) in combination with intensive insulin therapy for up to two years experienced significantly reduce HbA1c levels. However, it is important to note that prolonged exposure of nicotinamide has been reported to result in impaired pancreatic β-cell function and cell growth [1].

Another pathway ‘*Fc gamma R-mediated phagocytosis*’ is also relevant and one study indicated that chronic hyperglycemia was significantly associated with reduced phagocytosis via either FcγRs receptor in DM2 [2].

Pathway ‘*EGFR tyrosine kinase inhibitor resistance*’ also relevant as several clinical cases of the reversal of both type 1 and 2 diabetes mellitus (T1DM, T2DM) during protein tyrosine kinase (TKI) administration have been reported. Experimental *in vivo* and *in vitro* studies have elucidated some of the mechanisms behind this effect. For example, inhibition of Abelson tyrosine kinase (c-Abl) results in β cell survival and enhanced insulin secretion, while platelet-derived growth factor receptor (PDGFR) and epidermal growth factor receptor (EGFR) inhibition leads to improvement in insulin sensitivity. In addition, inhibition of vascular endothelial growth factor receptor 2 (VEGFR2) reduces the degree of islet cell inflammation (insulitis). Therefore, targeting several PTKs may provide a novel approach for correcting the pathophysiologic disturbances of diabetes [3].

**Subcutaneous Fat**

| **S. No.** | **Gene Set** | **Description** | **Size** | **Expect** | **Ratio** | **P Value** |
| --- | --- | --- | --- | --- | --- | --- |
| 1 | hsa03010 | Ribosome | 134 | 4.4599 | 3.1391 | 0.00013661 |
| 2 | hsa04142 | Lysosome | 123 | 4.0938 | 2.9313 | 0.00076529 |
| 3 | hsa04144 | Endocytosis | 244 | 8.1210 | 2.2165 | 0.0012402 |
| 4 | hsa04380 | Osteoclast differentiation | 128 | 4.2602 | 2.5820 | 0.0034739 |
| 5 | hsa03040 | Spliceosome | 133 | 4.4266 | 2.2591 | 0.013006 |
| 6 | hsa04973 | Carbohydrate digestion and absorption | 44 | 1.4644 | 3.4143 | 0.014771 |
| 7 | hsa04722 | Neurotrophin signaling pathway | 119 | 3.9607 | 2.2723 | 0.017417 |
| 8 | hsa05221 | Acute myeloid leukemia | 66 | 2.1967 | 2.7314 | 0.021965 |
| 9 | hsa05014 | Amyotrophic lateral sclerosis (ALS) | 51 | 1.6974 | 2.9456 | 0.026516 |
| 10 | hsa04926 | Relaxin signaling pathway | 130 | 4.3268 | 2.0801 | 0.029027 |

Pathway ‘*Osteoclast differentiation’* could be implicated in glucose homeostasis. Recently, an increasing number of studies have shown that bone is one type of endocrine organ which can regulate the energy metabolism. Obesity, high blood sugar levels and insulin resistance occurred in osteocalcin (OC) knockout mice. Insulin can promote osteoblasts to produce OC, and the bone regulates carbohydrate metabolism via OC. Osteoclast (a type of macrophage) is another type of cell in bone. One study showed that the resistin (adopcytoikine) expression was increased during osteoclast differentiation, and osteoclasts may affect glucose uptake in C2C12 cells. Osteoclasts may promote glucose uptake-related insulin resistance by secreting resistin. The inhibition on osteoclast formation may be a potential treatment strategy for insulin resistance or diabetes [4].

The implication of ‘*Neurotrophin signaling pathway*’ in metabolic disorder has been explored. Nerve growth factor (NGF) was the first neurotrophin described. NGF signaling has been noted in some of the immunoendocrine tissues most relevant to MetS development. Although there are several uncertainties about whether circulating NGF in MetS patients is up- or downregulated, the mechanistic evidence points to the relevance of local, rather than systemic, NGF concentrations for the pathophysiology of MetS, specifically by modulating the beta cell viability, GSIS, adipocyte metabolic profile, and inflammatory phenotype, and by influencing sex dimorphism in the development of MetS signs. Future research is needed to fully understand the changes in NGF signaling at each stage of MetS [5].

The pathway ‘*Relaxin signaling pathway*’ is relevant also. In the last decade, evidence has been accumulating that relaxin has major effects on the heart, blood vessels and the extracellular matrix within connective tissues. In particular, pertaining to the relationships between relaxin and diabetes, relaxin was shown to promote arterial and microvascular dilation, thereby increasing organ perfusion, counteract ischemic injury, improve adverse cardiac and vascular remodeling, and promote extracellular matrix turn-over, thereby exerting anti-fibrotic effects. Thus, relaxin could blunt or delay the vascular and organ complication of diabetes. Whether relaxin may also synergize with insulin to optimize blood glucose homeostasis remains an unconfirmed issue. However, there are clues in the literature which, if gathered, speak in favour of this perspective. Moreover, preliminary data suggest that exogenous relaxin administration may improve insulin sensitivity in diabetic patients [6].

**Visceral Fat**

| **S. No.** | **Gene Set** | **Description** | **Size** | **Expect** | **Ratio** | **P Value** |
| --- | --- | --- | --- | --- | --- | --- |
| 1 | hsa05226 | Gastric cancer | 148 | 3.0532 | 3.2752 | 0.00092449 |
| 2 | hsa04010 | MAPK signaling pathway | 295 | 6.0858 | 2.4647 | 0.0010729 |
| 3 | hsa01521 | EGFR tyrosine kinase inhibitor resistance | 79 | 1.6298 | 4.2951 | 0.0011573 |
| 4 | hsa04144 | Endocytosis | 244 | 5.0337 | 2.5826 | 0.0015275 |
| 5 | hsa05225 | Hepatocellular carcinoma | 167 | 3.4452 | 2.9026 | 0.0022989 |
| 6 | hsa05205 | Proteoglycans in cancer | 198 | 4.0847 | 2.6930 | 0.0025361 |
| 7 | hsa05214 | Glioma | 71 | 1.4647 | 4.0963 | 0.0033144 |
| 8 | hsa05206 | MicroRNAs in cancer | 150 | 3.0945 | 2.9084 | 0.0037334 |
| 9 | hsa04150 | mTOR signaling pathway | 151 | 3.1151 | 2.8891 | 0.0039016 |
| 10 | hsa05165 | Human papillomavirus infection | 339 | 6.9935 | 2.1448 | 0.0041478 |

Pathway ‘*MAPK signaling pathway’* is relevant also. Mitogen-activated protein kinase (MAPK) signalling occurs in response to almost any change in the extracellular or intracellular milieu that affects the metabolism of the cell, organ or the entire organism. MAPK-dependent signal transduction is required for physiological metabolic adaptation, but inappropriate MAPK signalling contributes to the development of several interdependent pathological traits, collectively known as metabolic syndrome. Metabolic syndrome leads to life-threatening clinical consequences, such as type 2 diabetes [7].

The mechanistic Target of Rapamycin (mTOR) coordinates eukaryotic cell growth and metabolism with environmental inputs including nutrients and growth factors. It forms the catalytic subunit of two distinct protein complexes, known as mTOR Complex 1 (mTORC1) and 2 (mTORC2). mTOR promotes adipocyte formation and lipid synthesis in response to feeding and insulin mTORC1 promotes adipogenesis and enhanced lipogenesis in cell culture and in vivo, consistent with adipocyte-specific raptor knock out (Ad-RapKO) mice displaying lipodystrophy and hepatic steatosis (Lee et al., 2016). Similarly, the loss of mTORC2 activity in adipocytes primarily results in insulin resistance due to reduced Akt activity (Kumar et al., 2010), but also in less lipid synthesis in part due to reduced expression of ChREBPβ, a master transcription factor for lipogenic genes (Tang et al., 2016). mTORC2 has also been shown to promote lipogenesis in the liver as well, suggesting a general role for mTORC2 in lipid synthesis (Hagiwara et al., 2012; Yuan et al., 2012) Thus, both mTORC1 and mTORC2 play important roles in multiple aspects of adipocyte function and lipid metabolism [8].

**References**

1. Maiese K. Nicotinamide: oversight of metabolic dysfunction through SIRT1, mTOR, and clock genes. Current neurovascular research. 2020 Dec 1;17(5):765-83.
2. Restrepo BI, Twahirwa M, Rahbar MH, Schlesinger LS. Phagocytosis via complement or Fc-gamma receptors is compromised in monocytes from type 2 diabetes patients with chronic hyperglycemia. PloS one. 2014 Mar 26;9(3):e92977.
3. Fountas A, Diamantopoulos LN, Tsatsoulis A. Tyrosine kinase inhibitors and diabetes: a novel treatment paradigm? Trends in Endocrinology & Metabolism. 2015 Nov 1;26(11):643-56.
4. Li X, Sun F, Lu J, Zhang J, Wang J, Zhu H, Gu M, Ma J. Osteoclasts may affect glucose uptake-related insulin resistance by secreting resistin. Diabetes, Metabolic Syndrome and Obesity. 2021 Jul 31:3461-70.
5. Samario-Román J, Larqué C, Pánico P, Ortiz-Huidobro RI, Velasco M, Escalona R, Hiriart M. NGF and its role in immunoendocrine communication during metabolic syndrome. International Journal of Molecular Sciences. 2023 Jan 19;24(3):1957.
6. Bani D, Pini A, Ka-Sheng Yue S. Relaxin, insulin and diabetes: an intriguing connection. Current Diabetes Reviews. 2012 Sep 1;8(5):329-35.
7. Gehart H, Kumpf S, Ittner A, Ricci R. MAPK signalling in cellular metabolism: stress or wellness?. EMBO reports. 2010 Nov;11(11):834-40.
8. Saxton RA, Sabatini DM. mTOR signaling in growth, metabolism, and disease. Cell. 2017 Mar 9;168(6):960-76.
